# Design and Implementation of an Informatics Infrastructure for Standardized Data Acquisition, Transfer, Storage, and Export in Psychiatric Clinical Routine: Feasibility Study

**DOI:** 10.2196/26681

**Published:** 2021-06-09

**Authors:** Rogério Blitz, Michael Storck, Bernhard T Baune, Martin Dugas, Nils Opel

**Affiliations:** 1 Institute of Medical Informatics University of Münster Münster Germany; 2 Department of Psychiatry University of Münster Münster Germany; 3 The Florey Institute of Neuroscience and Mental Health University of Melbourne Melbourne Australia; 4 Department of Psychiatry Melbourne Medical School University of Melbourne Melbourne Australia; 5 Institute of Medical Informatics Heidelberg University Hospital Heidelberg Germany; 6 Institute for Translational Psychiatry University of Münster Münster Germany; 7 Interdisciplinary Centre for Clinical Research of the Medical Faculty University of Münster Münster Germany

**Keywords:** medical informatics, digital mental health, digital data collection, psychiatry, single-source metadata architecture transformation, mental health, design, implementation, feasibility, informatics, infrastructure, data

## Abstract

**Background:**

Empirically driven personalized diagnostic applications and treatment stratification is widely perceived as a major hallmark in psychiatry. However, databased personalized decision making requires standardized data acquisition and data access, which are currently absent in psychiatric clinical routine.

**Objective:**

Here, we describe the informatics infrastructure implemented at the psychiatric Münster University Hospital, which allows standardized acquisition, transfer, storage, and export of clinical data for future real-time predictive modelling in psychiatric routine.

**Methods:**

We designed and implemented a technical architecture that includes an extension of the electronic health record (EHR) via scalable standardized data collection and data transfer between EHRs and research databases, thus allowing the pooling of EHRs and research data in a unified database and technical solutions for the visual presentation of collected data and analyses results in the EHR. The Single-source Metadata ARchitecture Transformation (SMA:T) was used as the software architecture. SMA:T is an extension of the EHR system and uses module-driven engineering to generate standardized applications and interfaces. The operational data model was used as the standard. Standardized data were entered on iPads via the Mobile Patient Survey (MoPat) and the web application Mopat@home, and the standardized transmission, processing, display, and export of data were realized via SMA:T.

**Results:**

The technical feasibility of the informatics infrastructure was demonstrated in the course of this study. We created 19 standardized documentation forms with 241 items. For 317 patients, 6451 instances were automatically transferred to the EHR system without errors. Moreover, 96,323 instances were automatically transferred from the EHR system to the research database for further analyses.

**Conclusions:**

In this study, we present the successful implementation of the informatics infrastructure enabling standardized data acquisition and data access for future real-time predictive modelling in clinical routine in psychiatry. The technical solution presented here might guide similar initiatives at other sites and thus help to pave the way toward future application of predictive models in psychiatric clinical routine.

## Introduction

### Scientific Background

Psychiatric disorders represent one of the leading causes of disability worldwide. In the challenge to provide advanced treatment and prevention strategies for psychiatric disorders, previous research has focused on better understanding of the neurobiological basis of affective disorders [1]. However, the translation of such findings into clinical application remains an unresolved problem up to now. For this reason, the focus of psychiatric research has shifted from sole neurobiological characterization at the group level toward the application of multivariate machine learning methods trained on multimodal data for individualized prediction of clinical outcomes [2,3]. Multivariate machine learning applications have been proven to be innovative and powerful tools in translational psychiatric research. In this regard, the successful utilization of machine learning algorithms for individualized predictions of treatment response [4-6], depression severity [7], disease risk [8], differential diagnosis [9,10], and relapse risk [11] has yielded the first promising results. However, up to now, several obstacles have prevented the successful transfer of individual predictive modeling to clinical routine application, as discussed in recent reviews [12-15]. In this regard, the gap between homogeneous well-characterized samples acquired in experimental studies [16] and heterogeneous unvalidated data from day-to-day clinical routine has proven to be a major obstacle in the translation of predictive models to clinical application. Hence, ecologically valid predictive models would require access to standardized real world data collected at the point of care [17].

Importantly, large-scale studies reporting the successful application of multivariate models trained on data from electronic health records (EHRs), including features such as diagnosis and procedures, laboratory parameters, and medications for the prediction of suicide risk or weight gain following antidepressant treatment have demonstrated the capacity and generalizability of predictive models trained on real-world data [18-20]. Further extension of EHRs via standardized collection of predictive variables such as known risk factors might further enhance the potential of this novel data entity for predictive analytics in psychiatry [21,22]. Standardized electronic collection of patient-reported outcomes that has previously been shown to improve clinical outcomes such as survival in patients with cancer represents another possibility to enrich EHR data. Similarly, combining data from EHRs with research data might provide new opportunities for the discovery and validation of psychiatric endophenotypes as demonstrated via recent validation of a polygenic risk score in a Danish population study [23]. However, future application of predictive models for personalized diagnostic and treatment requires their validation via clinical trials that, in turn, critically depend on the availability of the informatics infrastructure for the application of predictive models in routine care. The required informatics infrastructure should facilitate the acquisition of standardized real world data at the point of care, potential enrichment with patient-reported outcomes or research data, and subsequent access to data for clinicians and researchers. However, while these technical requirements are already available in selected clinical settings, for example, in the United States [24], they are up to now absent in the clinical working environment of psychiatry hospitals in many European countries. More concretely, ORBIS, the EHR system that is the market leader in Germany, Austria, and Switzerland, does not currently support standardized form metadata, clinical data, or annotated data sets. Our approach thus addresses the currently unmet need to (1) implement the technical requirements for standardized data acquisition and analysis in one of the most widely used EHR systems in Europe and (2) to specifically design a technical solution, including appropriate data collection routines, for the domain of clinical psychiatry.

This study aims to present the design and implementation of the technical requirements to address the aforementioned challenges with the ultimate goal of providing the basis for a successful future translation of predictive models to clinical application in psychiatric disorders. The implementation of the outlined technical solution will ultimately allow the evaluation of the potential of predictive models for the clinical management of psychiatric disorders under real-world conditions. In detail, we present the design and implementation of the informatics infrastructure, including technical solutions for (1) extension of the EHR via standardized electronic collection of patient-reported outcomes, (2) data transfer between EHRs and research databases, (3) pooling of EHRs and research data in a unified database, and (4) visual presentation of the analyses results in the EHRs. 

### Objective of This Study

The main objective of this study was the design and successful implementation of the informatics infrastructure required to train and validate predictive models in day-to-day clinical application in psychiatry as part of the SEED 11/19 study [25]. Our study consisted of the following steps in detail:

1. Implementation of standardized documentation forms in EHRs.
2. The set-up of an interface for direct data transfer between clinical documentation systems and a database for predictive analysis.
3. The set-up of a unified database that allows pooling of clinical data with further research data for predictive analysis.
4. Visual presentation of relevant data entities and results of predictive analysis in EHRs at the point of care.

## Methods

### Setting

The Münster University Hospital in Germany is a tertiary care hospital with 1457 beds and 11,197 staff who treated 607,414 patients (inbound and outbound) in 2019 [26]. The department for psychiatry and psychotherapy at the University Hospital treated 1341 cases in the study period from February 25, 2019 to July 31, 2020 (1042 cases in 2018 [27,28]). Validation was carried out by 25 doctors and 61 specialists from the health care sector.

### System Details

The EHR system ORBIS by Dedalus Healthcare is used at Münster University Hospital in more than 40 clinics and is the market leader in Germany, Austria, and Switzerland with over 1300 installations [29]. The EHR system has an 8700 GB Oracle database, 7938 users, and 1927 user sessions per day (status at July 2020) at Münster University Hospital. No standardized metadata form, clinical data, and annotated data sets are supported.

### Requirement Engineering

To address the study aims, the following requirements were identified through focus groups including physicians and researchers at Münster University Hospital in Germany.

1. Extension of the EHR via standardized data collection: At first sight, the widely established usage of electronic documentation systems in clinical routine might supplement the notion of a fast translation of predictive models. However, until now, the majority of clinical data is still acquired and stored in an unstructured way that cannot be directly used for predictive modeling. Extension of EHR data via standardized forms of data collection in routine care is therefore required to provide a sufficient database for the development of predictive models. Importantly, the technical solution should be flexible and allow to update the content of the collected EHR data. Content-wise, in an initial step, standardized extension of EHR data should include assessment of symptomatology in order to allow both patient stratification at baseline as well as outcome measurement following intervention. Furthermore, standardized assessment of known risk factors, including life events and sociodemographic data, appears meaningful.
2. Data transfer: Routine EHR data storage systems are usually strictly separated from research databases for safety reasons and hence are not directly accessible for predictive analyses. Training and validation of predictive models based on EHR data requires the set-up of interfaces and a database in which EHR data can be transferred and subsequently stored in a standardized way. In line with our study aim, the technical solutions should be scalable and allow data transfer in real time. EHR data transferred and stored in the database must be accessible for researchers in order to allow the development of predictive models.
3. Combination of EHRs and research data: Again, since routine EHR data storage systems are strictly separated from research databases, pooling of EHR and research data is not possible within state-of-the art EHR databases. Pooling EHR with research data in a unified database would allow the enrichment of predictive models trained on EHR data by adding already existing research data and furthermore to validate EHR data based on research data. To this end, in order to combine each patient’s EHR and research data, a unified scalable research database is needed that allows the integration of EHRs and research data acquired via experimental studies.
4. Presentation of standardized data within the EHR: Once collected, clinically useful standardized data as well as results of any analysis must be transferred back to the main EHR system in real time and presented to the clinician at the point of care.

### Solution Requirements

An informatics infrastructure enabling real-time clinical predictive modeling based on the single-source architecture was derived from the named requirements. Custom metadata must be supported. The Clinical Data Interchange Standards Consortium (CDISC) Operational Data Model (ODM) (version 1.3.2) was used as a flexible standard for exchange and archiving of metadata within the framework of clinical studies [30,31]. Mobile apps must be able to communicate with the architecture. Automatic data transfer into the database of the EHR system and from the EHR system to a research database was carried out via a communication server. ODM files were transported automatically to the database of the EHR system with Health Level 7 (HL7) messages [32]. NextGen Connect [33] was used as a communication server. HL7 version 2.5 and message type ORU^R01 were used. The plausibility and completeness of form data were validated by the clinical users.

### Analysis of Technical and Clinical Feasibility

The technical feasibility was demonstrated by the implementation of an infrastructure that enables clinical predictive modeling in real time. Java version 1.8.0_181 [34], JavaScript ECMAScript 6 [35], TypeScript version 3.7.2 [36], and the proprietary language of the EHR system were used as programming languages. MongoDB Java Drivers version 3.9.1 [37] and Json-lib version 2.4 [38] were used as third-party libraries. MongoDB version 4.2.3 [39] was used as a research database, Docker version 19.03.13 [40] for operating system–level virtualization, and Red Hat Enterprise version 7.8 [41] as research server. The clinical feasibility was determined by piloting the architecture in the clinic for psychiatry and psychotherapy and for a prospective analysis of the clinical documentation forms used. The clinical users of the system were 25 doctors and 61 health care sector specialists. The stakeholder of the study at Münster University Hospital is the Institute for Translational Psychiatry, Department of Psychiatry. Evaluation began on February 25, 2019 and ended on July 31, 2020. EHR data from daily clinical routine (eg, laboratory data, diagnostic codes) and self-reports/patient-reported outcomes that were experimentally collected as an extension of the clinical routine documentation as part of the SEED research project were examined. The following evaluation criteria were analyzed: (1) measurement of data completeness in the created documentation forms, (2) measurement of data completeness in the research database, (3) monitoring of system stability, and (4) monitoring of data transfer. SPSS Statistics version 25 (IBM Corp) [42] was used for descriptive data analysis. Adobe Photoshop version 11.0 [43] and Microsoft Visio version 16.0.4849.1000 [44] were used to depict the workflow.

## Results

### System Architecture

**Figure 1:**
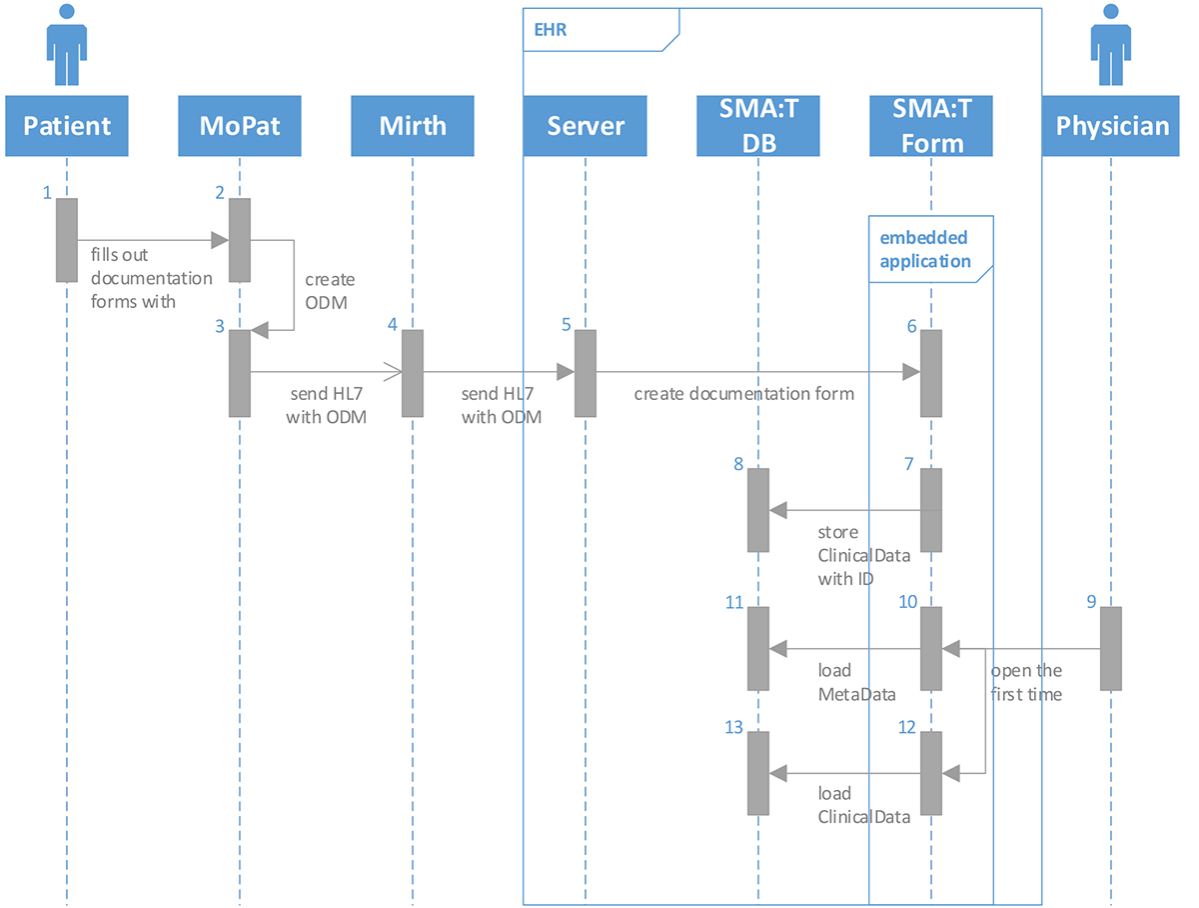
Unified Modeling Language sequence diagram of the data collection workflow. In process steps 1-3, the patient completes the forms and sends data to the communication server. In process steps 4-8, the communication server sends data to the electronic health record system and creates a blank documentation form. This form is populated with imported data. In process steps 9-13, SMA:T creates the documentation form with metadata and imported data. EHR: electronic health record; HL7: Health Level 7; MoPat: Mobile Patient Survey; ODM: operational data model; SMA:T: Single-source Metadata ARchitecture Transformation.

**Figure 2:**
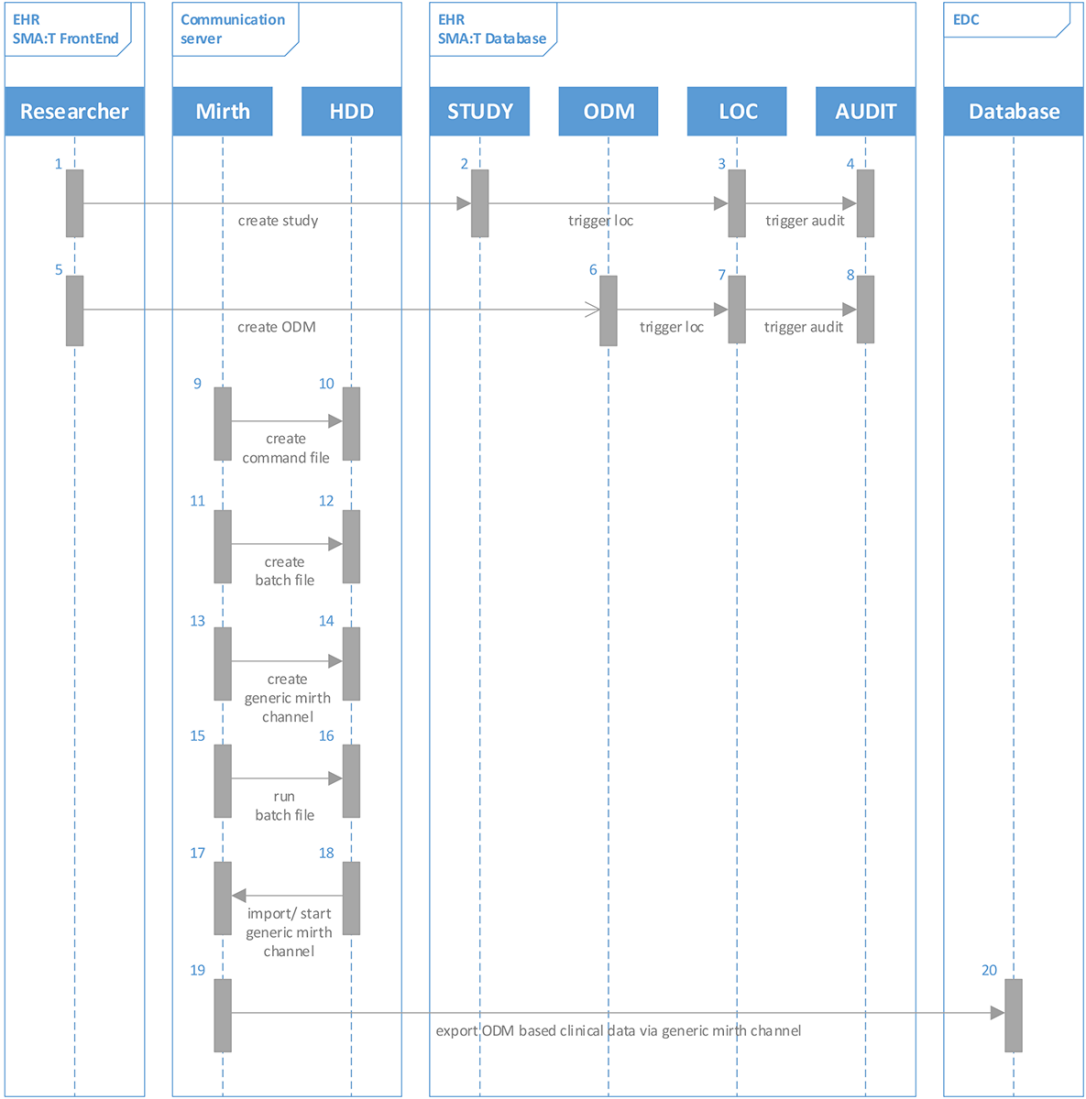
Unified Modeling Language sequence diagram of the data extraction workflow. In process steps 1-8, a study query is created with SMA:T and a generic operational data model file is saved in the database of the electronic health record system. In process steps 9-18, a generic Mirth Channel is created based on the study query. In process steps 19-20, data points are automatically extracted from the electronic health record system and transferred to the study database using operational data model standard format. EDC: electronic data capture; EHR: electronic health record; HDD: Hard Disc Drive; HL7: Health Level 7; LOC: Lines Of Code; MoPat: Mobile Patient Survey; ODM: operational data model; SMA:T: Single-source Metadata ARchitecture Transformation.

**Figure 3:**
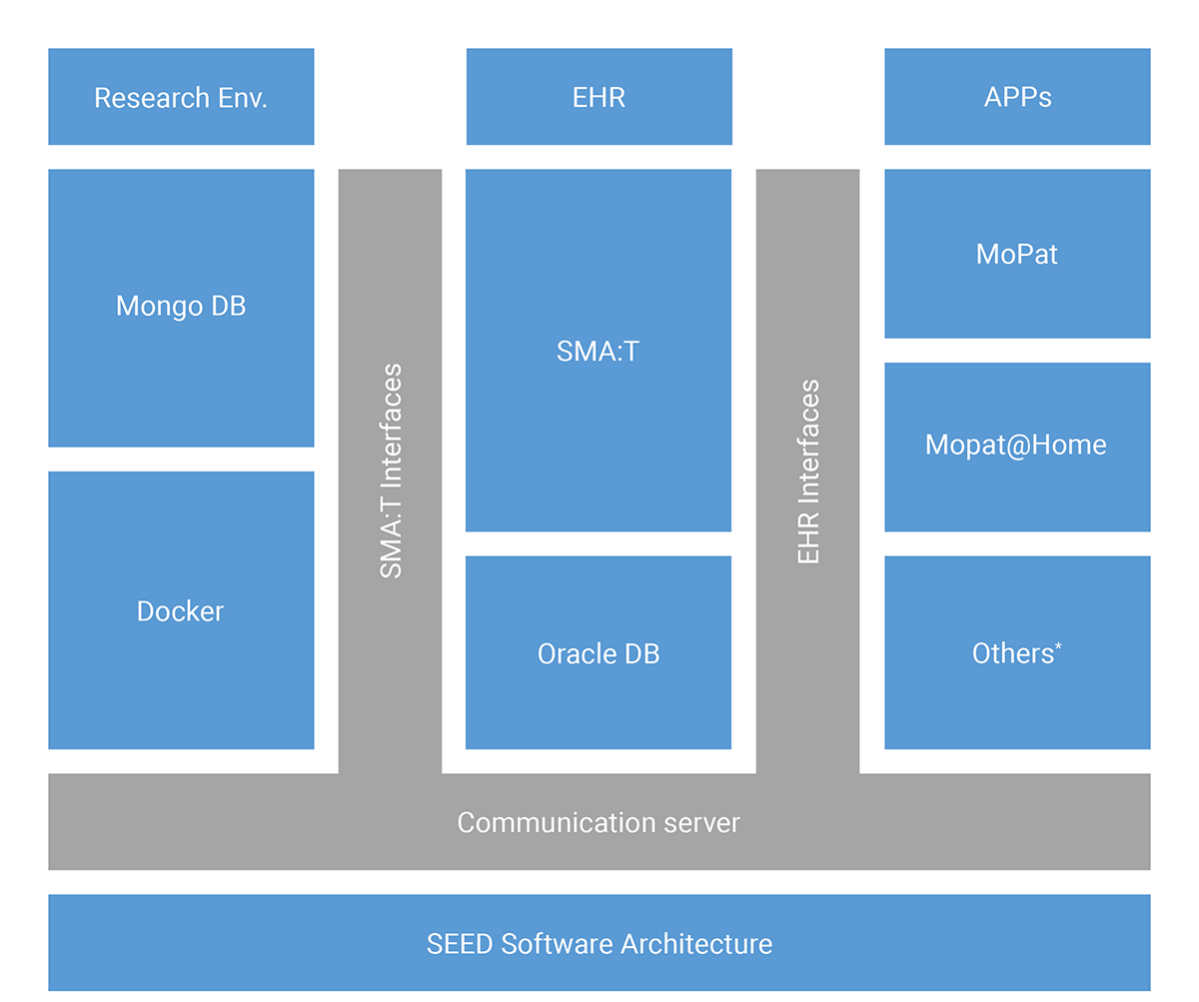
SEED software architecture of the Münster University Hospital. EHR: electronic health record; MoPat: Mobile Patient Survey; SMA:T: Single-source Metadata ARchitecture Transformation; *supports custom applications.

The Single-source Metadata ARchitecture Transformation (SMA:T) was used as the software architecture [45]. SMA:T is an extension of the EHR system of the Münster University Hospital and uses module-driven software development [46] to generate standardized applications and interfaces. Every SMA:T form has a generic built-in interface for exchanging standardized data. Embedded applications [45] were used as the application type. These are linked to an ODM file in the EHR database, from which a documentation form is generated. All metadata and clinical data are available in the ODM developed by CDISC version 1.3.2. Patient-reported outcomes are recorded via Mobile Patient Survey (MoPat) [47,48] on mobile devices (generation 6 iPads) and via the web application Mopat@home (a modified version of the tablet-based web app MoPat) [49] for follow-up assessments following discharge from inpatient treatment. Collected data are transferred to the communication server via an HL7 message and from there to the database of the EHR system. Data are sent in the OBX-5 segment of the HL7 message. SMA:T provides database storage. A reference to the imported clinical data is saved. Clinical data are automatically inserted by SMA:T when the documentation form is opened for the first time. A unique ID from the HL7 header is used for this purpose. Each ID is linked to an imported clinical record. The structure of the architecture is shown in Figure 1. Data transfer to the research database takes place via the researcher module from SMA:T. This provides a front end to the EHR system and an extension of the communication server for data transfers. Both prospective and retrospective standardized data exports of EHR data points are supported, specifically, vital signs, laboratory data, medication data, and administrative data. Each data export can be customized by individual parameters. The following parameters are supported: name of data export, export interval, database query, destination parameters for electronic data capture systems, or research databases. MongoDB and RedCap [50] are currently provided as destination templates in the EHR system. The destination portfolio can easily be expanded with interface functions of SMA:T. The research database is embedded in a Docker container of a virtualized Red Hat Enterprise Linux server. The data flow from EHR to electronic data capture is shown in Figure 2. The software architecture is shown in Figure 3.

### System Implementation

The implementation of the architecture is divided into 4 areas: data collection, data transfer, data storage, and data visualization. Agile methods were used for Project Life Cycle and Development Cycle [51].

#### Data Collection

**Figure 4:**
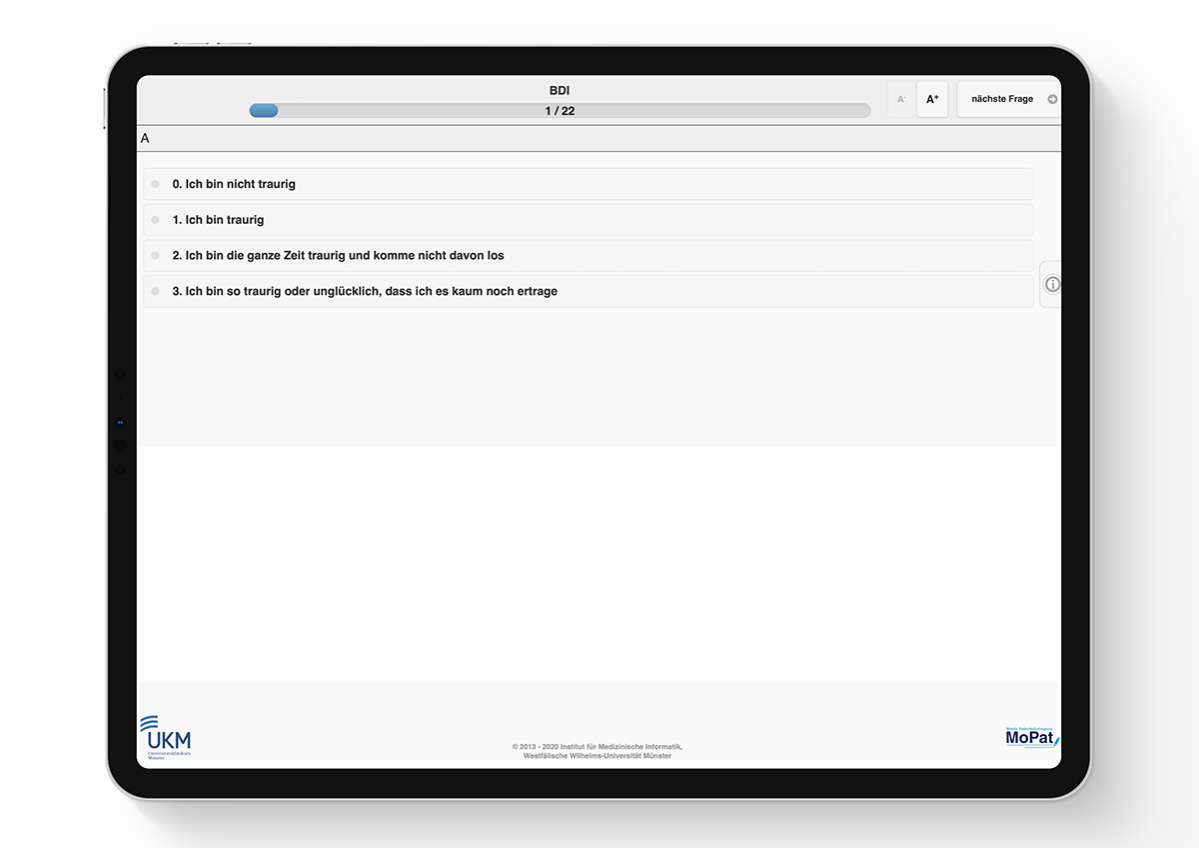
One item of Beck Depression Inventory presented in the MoPat app (clinic for psychiatry and psychotherapy at Münster University Hospital).

SMA:T provides 2 options for data collection, namely, the EHR system in clinical routine and dedicated web applications. Data input via web applications can be designed freely. In this study, EHR data generated as part of clinical routine documentation comprised, among others, laboratory data, medication, information on diagnosis, time of admission, and length of stay and are presented in detail in Table 1. MoPat [47,48] was selected for the collection of patient-reported outcomes. After input of the patient case ID, staff handed the patient an iPad with the MoPat app. Patients were then guided through a series of documentation forms comprising different questionnaires and they entered data on the mobile device (Figure 4). The iPad was then returned to the medical staff. Further details regarding the collection of patient-reported outcomes during inpatient treatment have previously been described [25]. In brief, the self-reports applied in this study are based on well-established questionnaires and scales in the domain of psychiatry and clinical psychology. In addition, to the retention of single item information, sum scales were calculated based on the recommendations provided in the original manuals and references [52-58]. In addition, Mopat@home was used for the collection of patient-reported outcomes following discharge. To this end, patients were sent an email, which provided a link to a website in which the above referenced questionnaires were presented and could be filled out [49].

**Table 1:** Research documentation used in the Department of Psychiatry.

Name of the documentation form	Items
SEED ClinicalData Admission Date & Time	4
SEED ClinicalData Classification	2
SEED ClinicalData Diagnosis-Related Groups/Diagnosis	3
SEED ClinicalData Electroconvulsive Therapy	11
SEED ClinicalData Laboratory Assessments	7
SEED ClinicalData Medication	5
SEED ClinicalData Patient	4
SEED ClinicalData Vital Signs	3

#### Data Transfer

SMA:T provides 2 types of data transfer in the present scenario, that is, data transfer into the EHR system and transfer into the electronic data capture system. MoPat sends data to the EHR system via the communication server of the University Hospital. Data are saved in the ClinicalData structure of the ODM format. The ODM document is embedded in an HL7 message. Each HL7 message creates a form in the EHR system. The header of the HL7 message determines which form is automatically created. Data transfer to the electronic data capture takes place via SMA:T interfaces. Both retrospective and prospective data exports in real time are supported. When a study query was activated via the EHR frontend, metadata and corresponding structured query language statements were read by the SMA:T extension of the communication server. SMA:T uses its code library and channel framework to generate unique Mirth channels. These send a database query to the EHR system and transfers the output directly to the electronic data capture system. Both metadata (clinical documentation form) and clinical patient data are provided by SMA:T in the ODM format. Data records are combined into an ODM document. In this study, SMA:T converts the resulting XML-based ODM document into JavaScript Object Notation format [59] (JODM format [60]). The JavaScript Object Notation schema [61] of JODM [60] is open source and currently limited to Study and ClinicalData nodes, including all subnodes of the ODM in version 1.3.2.

#### Data Storage

Data storage addresses metadata and clinical data. Metadata of clinical documentation forms are stored centrally in the SMA:T database. The SMA:T database model is part of the EHR database model. Metadata and clinical data are available in the ODM format. MoPat also supports ODM format; therefore, the same data model can be used for both systems. Clinical data are clearly identified by unique object identifiers and the associated object identifier on the documentation form.

#### Data Presentation

Usability principles were applied to visualize data [62-64]. A one-column layout was implemented according to the requirements of the 10 web form design guidelines [65]. Those forms are displayed via SMA:T within the EHR system (see Multimedia Appendix 1 and Multimedia Appendix 2). SMA:T supports item-based real-time notifications as well as centralized notification services to display analysis results in real time.

### Technical and Clinical Feasibility

As part of the study, 11 standardized documentation forms with 202 items were created for the clinic for psychiatry and psychotherapy (Table 2): Beck Depression Inventory [66], Big Five Inventory (BFI-2-S) [67], Big Five Inventory (BFI-2-XS) [68], Childhood Trauma Questionnaire [69], Family Mental History [70], Hamilton Depression Scale [71], Narcissistic Admiration and Rivalry Questionnaire [72], Symptom Checklist-90 Somatization Scale [73], sociodemographic questionnaire [74], questions on individual disease course [75], and questions on somatic comorbidities [76]. Data models without license restrictions are available in the portal of medical data models. A documentation form is a document from the EHR system (see Multimedia Appendix 1) and consists of several items. An item consists of an input field and the associated label. For example, 1 item from Multimedia Appendix 1 is the drop-down box labeled A; 5866 instances were created by the patients and automatically transferred to the EHR system of the Münster University Hospital without errors. An instance is a form created by a user; 412 cases from 317 patients were processed by 86 users (Table 3). A case is defined as an inpatient stay or an outpatient visit to a hospital or clinic. Of the 123 of the medical staff of the clinic for psychiatry and psychotherapy, 86 (69.9%) worked with those documentation forms. The data quality could be improved by the ODM. Metadata was a critical step in building a generic and automated workflow. All items are now provided with a unique object identifier, have a typing of the data types, and a code list for converting text into numeric values. Automatic generation of documentation forms was accepted in routine clinical use. Standardized data transfer from the communication server into the EHR system was completed without error. It was possible to display all items (n=202) from ODM structures in full by using the generic workflow. Clinical data from 317 patients was stored in the EHR database; 96.7% (4360/4509) of the scores could be calculated and transferred into the EHR system (Beck’s Depression Inventory [52,53], Big Five Inventory [54], Childhood Trauma Questionnaire [55], Hamilton Depression Scale [56], Narcissistic Admiration and Rivalry Questionnaire [57], and Symptom Checklist-90 Somatization Scale [58]) (Table 4), and 111,842 items were completed by patients on mobile devices (Table 5). Approximately 99.3% (91,329/91,974) of forms with scores (91,974 items/645 uncompleted items) were completed and 88.1% (12,617/14,318) of forms without scores (14,318 items/1701 uncompleted items) were completed. The validity of the acquired data on depressive symptomatology was already analyzed in a feasibility study [25]. Eight standardized documentation forms with 39 items were created for the retrospective data export (Table 1): SEED ClinicalData Admission Date & Time [77], SEED ClinicalData Classification [78], SEED ClinicalData Diagnosis-Related group/Diagnosis [79], SEED ClinicalData electroconvulsive therapy [80], SEED ClinicalData Laboratory Assessments [81], SEED ClinicalData Medication [82], SEED ClinicalData Patient [83], and SEED ClinicalData Vital Signs [84]. A total of 96,323 instances of vital signs, laboratory data, medication data, and administrative data could be automatically transferred from the EHR system to the research database (Table 6). Retrospective ODM-based data export worked correctly without technical errors, and 585 instances were created by the patients with Mopat@home and transferred to the research database via SMA:T (Table 7).

**Table 2:** Routine documentation used in the Department of Psychiatry.

Name of the documentation form (n=11)	Items (n=202)
Beck Depression Inventory	23
Big Five Inventory (BFI-2-S)	35
Big Five Inventory (BFI-2-XS)	20
Childhood Trauma Questionnaire	34
Family Mental History	14
Hamilton Depression Scale	25
Narcissistic Admiration and Rivalry Questionnaire	9
Symptom Checklist-90 Somatization Scale	14
Sociodemographic questionnaire	5
Questions on individual disease course	18
Questions on somatic comorbidities	5

**Table 3:** Number of instances created for each documentation form: the counts of patients, patient cases, and users are shown.

Name of the documentation form	Cases	Patients	Instances	Users
Beck Depression Inventory	380	307	1266	50
Big Five Inventory (BFI-2-S)	358	303	559	25
Big Five Inventory (BFI-2-XS)	258	217	692	19
Childhood Trauma Questionnaire	313	303	354	33
Family Mental History	315	305	343	31
Hamilton Depression Scale	350	296	516	42
Narcissistic Admiration and Rivalry Questionnaire	357	302	558	20
Symptom Checklist-90 Somatization Scale	360	303	564	18
Sociodemographic questionnaire	315	305	344	26
Questions on individual disease course	315	305	342	26
Questions on somatic comorbidities	313	303	328	10

**Table 4:** Data quality of patient-based documentation regarding score calculation.

Name of the documentation form	Instances	Scores	Missing data^a^
Beck Depression Inventory	1266	1238	28
Big Five Inventory (BFI-2-S)	559	540	19
Big Five Inventory (BFI-2-XS)	692	656	36
Childhood Trauma Questionnaire	354	320	34
Hamilton Depression Scale	516	502	14
Narcissistic Admiration and Rivalry Questionnaire	558	550	8
Symptom Checklist-90 Somatization Scale	564	554	10

^a^Missing data frequency is determined by missing data entries.

**Table 5:** Data on the completeness of the documentation forms.^a^

Name of the documentation form	Items	Completed items	Uncompleted items
Beck Depression Inventory	29,118	29,015	103
Big Five Inventory (BFI-2-S)	19,565	19,519	46
Big Five Inventory (BFI-2-XS)	13,840	13,739	101
Childhood Trauma Questionnaire	12,036	11,985	51
Family Mental History	4802	4354	448
Hamilton Depression Scale	12,384	12,076	308
Narcissistic Admiration and Rivalry Questionnaire	5031	5012	19
Symptom Checklist-90 Somatization Scale	7896	7879	17
Sociodemographic questionnaire	1720	1715	5
Questions on individual disease course	6156	5453	703
Questions on somatic comorbidities	1640	1095	545

^a^In this context, completeness means that the documentation form contains values in all data points.

**Table 6:** Number of retrospectively transferred research documentation forms (electronic health record to electronic data capture).^a^

Name of the documentation form	Instances in electronic health records
SEED ClinicalData Admission Date & Time	245
SEED ClinicalData Classification	8260
SEED ClinicalData Diagnosis-Related Groups/Diagnosis	1163
SEED ClinicalData Electroconvulsive therapy	452
SEED ClinicalData Laboratory Assessments	22,886
SEED ClinicalData Medication	14,244
SEED ClinicalData Patient	245
SEED ClinicalData Vital Signs	48,828

^a^Electronic health record data were extracted with generic study queries in the Single-source Metadata ARchitecture Transformation system.

**Table 7:** Number of instances created with Mopat@home for each documentation form.

Name of the documentation form	Instances
Beck Depression Inventory	65
Big Five Inventory (BFI-2-S)	64
Childhood Trauma Questionnaire	65
Family Mental History	65
Narcissistic Admiration and Rivalry Questionnaire	64
Symptom Checklist-90 Somatization Scale	66
Sociodemographic questionnaire	66
Questions on individual disease course	65
Questions on somatic comorbidities	65

## Discussion

### Answers to the Study Questions

The aim of this study was the design and implementation of an informatics infrastructure enabling standardized data acquisition at the point of care and subsequent accessibility of clinical data for analytic purposes, which is required for future application of predictive models in day-to-day clinical routine in psychiatry. In this study, we have shown the overall technical feasibility of the implemented solution. Standardized documentation forms were implemented to extend EHR data domains and to improve data quality in the EHR system. An automated transfer of data into the EHR system and the research database was implemented, thus enabling the pooling of EHR data with already existing research data from ongoing cohort studies. This system was accepted by clinical staff from the Department of Psychiatry of Münster University Hospital in Germany. Widespread use of documentation forms could be demonstrated. Standardized electronic data collection in the EHR at the point of care was successfully implemented. The latter solution can similarly be applied for the presentation of results from predictive models.

### Strengths and Weaknesses of This Study

The major strengths of this study are standardized acquisition, transfer, storage, and export of data in real time with a generic informatics infrastructure. This system fulfills the prerequisites for future predictive modelling in clinical routine in psychiatry [85-87]. Standardized data transfer in ODM format provides scalability in the context of complex medical data structures. The Define-XML standard, an extension of the ODM standard, is mandated by regulatory authorities such as Food and Drug Administration for metadata [88]. Compliance with regulatory standards is the major advantage of our infrastructure regarding future clinical studies. The data format had to be converted due to the research database, which is a limitation. MongoDB was chosen for rapid analysis of large amounts of data in previous work [89]. Standardized automatic data transfer into research databases was possible for both retrospective and prospective research questions. The data of the EHR system was responsible for the number of documentation forms for the retrospective export. Data export can be configured centrally from the EHR system in compliance with local data protection regulations. Our approach is scalable because ORBIS EHR systems are used in more than 1300 hospitals in Germany, Austria, and Switzerland. The evaluation concentrated on technical and clinical feasibility. Limitations include the lack of elaborated standardized evaluations of the user experience of the system by clinical staff. Moreover, further evaluation is necessary in order to assess the sustainable benefit in everyday clinical practice. Although the feasibility and acceptability of the implemented data input interface has been demonstrated in a recent publication [24] and the wide-spread use of the implemented data presentation format in the EHR indicates acceptability, it appears important to note that no further feedback from clinicians (ie, in the form of structured interviews or questionnaires) has been acquired, which limits the informative value regarding user satisfaction. This important issue should therefore be addressed by future works based on elaborated user feedback. Of note, the projected acquisition of data from several hundred cases per year based on our set-up results in a database of modest scale was comparable to that by successfully established deep learning models in other fields of medicine [90]. Yet, it appears important to take into account that that the current state-of-the-art machine learning approaches in psychiatric research are based on cohorts with smaller sample sizes that were acquired over a period of multiple years [91-93]. The present initiative that aims to train predictive models on data from clinical routine documentation thus offers a perspective to significantly increase sample sizes in machine learning research in psychiatry. The training of predictive models as well as their validation in clinical applications is not within the scope of the this study but will be the focus of subsequent work building on the technical infrastructure outlined in this study. Importantly, as our standardized data acquisition protocol covers established risk factors and symptom profiles that have in part already been successfully used for predictive analytics in psychiatric cohort studies [91,93], it appears reasonable to assume their predictive validity for the intended prediction of symptom trajectories and functional outcomes in future work.

### Results in Relation to Other Studies

Through our study, we extend a previous line of research on predictive modeling based on EHR data. While previous studies have demonstrated empirical evidence for the predictive validity of EHR data in psychiatric use cases [18-20], to the best of our knowledge, our study is the first to not only report on the design but also on the successful implementation and technical feasibility of the informatics infrastructure for standardized acquisition, transfer, storage, and access of real world data for analytic purposes in psychiatric care, which is the basic requirement for the application and validation of predictive models in future clinical studies. Although we are not aware of any other study that has reported successful implementation of a comparable informatics infrastructure in psychiatric clinical routine, several preliminary reports should be taken into account. Complementary to the work presented in this study, Khalilia et al [94] described a Fast Healthcare Interoperability Resources (FHIR) web modeling service that was tested on a pilot intensive care unit dataset. A multi-source approach was used. No binding standard is used for clinical studies; instead, the standard Observational Medical Outcomes Partnership Common Data Model was applied [95] and an FHIR server and database are required for this system, which might limit potential implementations at multiple sites, considering that many EHR systems currently do not yet use an FHIR server. Of note, we are aware of several large-scale efforts aiming to translate predictive models into psychiatric practice [96] that, once implemented, might serve as a future base for comparison of system stability and performance. Importantly, the presented infrastructure represents a flexible solution that allows compatibility with existing initiatives and concepts of data standardization such as the Common Data Elements repository of the National Institutes of Health [97]. The choice of the ODM as the data standard implies the automatic provision of a metadata provider for each item. Thus, data points can be enriched with additional codes based on standards such as the Systematized Nomenclature of Medicine Clinical Terms or the Unified Medical Language System [98,99]. The integration takes place via the alias node or the SMA:T schema extension of the ODM. This makes it possible to enrich the survey data with additional metadata. International standardizations are hence compatible with the operating data model based on a 1-1 mapping of item definition nodes.

### Generalizability of This Study

The informatics infrastructure for standardized data acquisition, transfer, storage, and export in real time for future predictive modelling outlined in this study is an important step in the complex process toward the implementation of machine learning and clinical decision support solutions in routine care. Our study shows that this approach is technically feasible. Owing to the standardization, this concept is also scalable for other medical areas. Data warehouse applications of a heterogeneous hospital landscape can be implemented with this software architecture. In addition to local artificial intelligence applications, multi-site implementations of the architecture could also transfer pseudonymized data points into a global predictive model. The implementation of national and international predictive models in medicine would be possible.

### Future Work

Artificial intelligence systems rely on high-quality data. In the future, artificial intelligence applications might send real-time evaluations directly back into the EHR system. Clinical staff could access and respond to calculated predictions. Selected data will be provided in a modular dashboard. Medical device regulation needs to be taken into account for implementation of such systems. Direct data transfer back from the clinic would be possible. Real-time adjustments of the prediction models would thus be possible. Standardization of clinical routine documentation via SMA:T can provide high-quality structured data points. It is planned to augment this database with further research data from existing cohort studies, for example, covering neuroimaging and genetic data. Specific prediction models can be trained in this way with the same architecture. Generic model pipelines can be set up. Model clusters can be set up to answer complex medical questions. Basically, SMA:T forms a solid technical infrastructure for the implementation of artificial intelligence solutions in medicine. Scheme extensions of the ODM standard can be implemented to optimize communication between systems. Observational and interventional studies are warranted to evaluate the predictive validity of machine learning models in psychiatric routine. For multi-center studies, SMA:T needs to be reimplemented in the respective EHR environments to process CDISC ODM files. A software blueprint is available [45]. If SMA:T and MoPat are already in use, the architecture can be set up within a short time frame of approximately 1 week. The generic concept of the architecture enables the reuse of our data models, database queries, and server architecture. Retrospective database queries might have to be reimplemented in the EHR environments. The necessary data can be used from our repository on GitHub [100]. Another important consideration is the potential future enrichment of EHR data with mobile assessments, including ecological momentary assessments and passive sensor data derived from smartphones. Recent reports on successful real-time prediction of depressive symptoms based on ecological momentary assessment data supplement this notion [101]. Thus, future studies should explore technical solutions that allow data transfer between EHRs and patients’ smartphones. Future work will evaluate the predictive potential of the acquired data entities by training and validating machine learning models for an individual level prediction of treatment response, functional outcome, and depression relapse. In accordance with findings from previous machine learning approaches in psychiatric cohort studies, in a first step, well-established predictive algorithms such as support vector machines will be trained on features covering risk and symptom profiles, sociodemographic variables, medication, and treatment history [7,91,93]. Yet importantly, as opposed to previous cohort studies, the technical infrastructure outlined in this study will allow to train and validate predictive models in naturalistic patient samples in routine care.

### Conclusions

The presented informatics infrastructure enabling standardized data acquisition, transfer, storage, and export in real time for future predictive modelling in clinical routine in psychiatry is technically feasible. The outlined architecture provides a technical basis for the application, first and foremost, and the validation of clinical decision support systems and artificial intelligence applications in clinical studies.

## Appendix

Multimedia Appendix 1 Beck Depression Inventory documentation form created with Single-source Metadata ARchitecture Transformation.

Multimedia Appendix 2 Symptom Checklist-90 Somatization Scale documentation form created with Single-source Metadata ARchitecture Transformation.

## Abbreviations

CDISC: Clinical Data Interchange Standards Consortium
EHR: electronic health record
FHIR: Fast Healthcare Interoperability Resources
HL7: Health Level 7
MoPat: Mobile Patient Survey
ODM: operational data model
SMA:T: Single-source Metadata ARchitecture Transformation

## References

[1] Schmaal L, Hibar D, Sämann P, et al (2017). Cortical abnormalities in adults and adolescents with major depression based on brain scans from 20 cohorts worldwide in the ENIGMA Major Depressive Disorder Working Group. Molecular Psychiatry volume 22.

[2] Hahn T, Marquand AF, Ehlis A, Dresler T, Kittel-Schneider S, Jarczok TA, Lesch K, Jakob PM, Mourao-Miranda J, Brammer MJ, Fallgatter AJ (2011). Integrating neurobiological markers of depression. Arch Gen Psychiatry.

[3] Huys QJM, Maia TV, Frank MJ (2016). Computational psychiatry as a bridge from neuroscience to clinical applications. Nat Neurosci.

[4] Chekroud AM, Zotti RJ, Shehzad Z, Gueorguieva R, Johnson MK, Trivedi MH, Cannon TD, Krystal JH, Corlett PR (2016). Cross-trial prediction of treatment outcome in depression: a machine learning approach. Lancet Psychiatry.

[5] Koutsouleris N, Meisenzahl EM, Borgwardt S, Riecher-Rössler Anita, Frodl T, Kambeitz J, Köhler Yanis, Falkai P, Möller Hans-Jürgen, Reiser M, Davatzikos C (2015). Individualized differential diagnosis of schizophrenia and mood disorders using neuroanatomical biomarkers. Brain.

[6] Redlich R, Opel N, Grotegerd D, Dohm K, Zaremba D, Bürger Christian, Münker Sandra, Mühlmann Lisa, Wahl P, Heindel W, Arolt V, Alferink J, Zwanzger P, Zavorotnyy M, Kugel H, Dannlowski U (2016). Prediction of Individual Response to Electroconvulsive Therapy via Machine Learning on Structural Magnetic Resonance Imaging Data. JAMA Psychiatry.

[7] Kessler RC, van Loo HM, Wardenaar KJ, Bossarte RM, Brenner LA, Cai T, Ebert DD, Hwang I, Li J, de Jonge P, Nierenberg AA, Petukhova MV, Rosellini AJ, Sampson NA, Schoevers RA, Wilcox MA, Zaslavsky AM (2016). Testing a machine-learning algorithm to predict the persistence and severity of major depressive disorder from baseline self-reports. Mol Psychiatry.

[8] Opel N, Zwanzger P, Redlich R, Grotegerd D, Dohm K, Arolt V, Heindel W, Kugel H, Dannlowski U (2015). Differing brain structural correlates of familial and environmental risk for major depressive disorder revealed by a combined VBM/pattern recognition approach. Psychol. Med.

[9] Koutsouleris N, Meisenzahl EM, Borgwardt S, Riecher-Rössler Anita, Frodl T, Kambeitz J, Köhler Yanis, Falkai P, Möller Hans-Jürgen, Reiser M, Davatzikos C (2015). Individualized differential diagnosis of schizophrenia and mood disorders using neuroanatomical biomarkers. Brain.

[10] Redlich R, Almeida JJR, Grotegerd D, Opel N, Kugel H, Heindel W, Arolt V, Phillips ML, Dannlowski U (2014). Brain morphometric biomarkers distinguishing unipolar and bipolar depression. A voxel-based morphometry-pattern classification approach. JAMA Psychiatry.

[11] Cearns M, Opel Nils, Clark Scott, Kaehler Claas, Thalamuthu Anbupalam, Heindel Walter, Winter Theresa, Teismann Henning, Minnerup Heike, Dannlowski Udo, Berger Klaus, Baune Bernhard T (2019). Predicting rehospitalization within 2 years of initial patient admission for a major depressive episode: a multimodal machine learning approach. Transl Psychiatry.

[12] Hirschtritt ME, Insel TR (2018). Digital Technologies in Psychiatry: Present and Future. Focus (Am Psychiatr Publ).

[13] Hsin H, Fromer Menachem, Peterson Bret, Walter Collin, Fleck Mathias, Campbell Andrew, Varghese Paul, Califf Robert (2018). Transforming Psychiatry into Data-Driven Medicine with Digital Measurement Tools. NPJ Digit Med.

[14] Rutledge RB, Chekroud AM, Huys QJ (2019). Machine learning and big data in psychiatry: toward clinical applications. Curr Opin Neurobiol.

[15] Torous J, Baker JT (2016). Why Psychiatry Needs Data Science and Data Science Needs Psychiatry: Connecting With Technology. JAMA Psychiatry.

[16] Zimmerman Mark, Clark Heather L, Multach Matthew D, Walsh Emily, Rosenstein Lia K, Gazarian Douglas (2015). Have Treatment Studies of Depression Become Even Less Generalizable? A Review of the Inclusion and Exclusion Criteria Used in Placebo-Controlled Antidepressant Efficacy Trials Published During the Past 20 Years. Mayo Clin Proc.

[17] Skyttberg N, Vicente J, Chen R, Blomqvist H, Koch S (2016). How to improve vital sign data quality for use in clinical decision support systems? A qualitative study in nine Swedish emergency departments. BMC Med Inform Decis Mak.

[18] Barak-Corren Y, Castro VM, Javitt S, Hoffnagle AG, Dai Y, Perlis RH, Nock MK, Smoller JW, Reis BY (2017). Predicting Suicidal Behavior From Longitudinal Electronic Health Records. Am J Psychiatry.

[19] Barak-Corren Y, Castro VM, Nock MK, Mandl KD, Madsen EM, Seiger A, Adams WG, Applegate RJ, Bernstam EV, Klann JG, McCarthy EP, Murphy SN, Natter M, Ostasiewski B, Patibandla N, Rosenthal GE, Silva GS, Wei K, Weber GM, Weiler SR, Reis BY, Smoller JW (2020). Validation of an Electronic Health Record-Based Suicide Risk Prediction Modeling Approach Across Multiple Health Care Systems. JAMA Netw Open.

[20] Blumenthal SR, Castro VM, Clements CC, Rosenfield HR, Murphy SN, Fava M, Weilburg JB, Erb JL, Churchill SE, Kohane IS, Smoller JW, Perlis RH (2014). An electronic health records study of long-term weight gain following antidepressant use. JAMA Psychiatry.

[21] Simon GE (2019). Big Data From Health Records in Mental Health Care: Hardly Clairvoyant but Already Useful. JAMA Psychiatry.

[22] Weissman MM, Pathak J, Talati A (2020). Personal Life Events-A Promising Dimension for Psychiatry in Electronic Health Records. JAMA Psychiatry.

[23] Musliner KL, Mortensen PB, McGrath JJ, Suppli NP, Hougaard DM, Bybjerg-Grauholm J, Bækvad-Hansen Marie, Andreassen O, Pedersen CB, Pedersen MG, Mors O, Nordentoft M, Børglum Anders D, Werge T, Agerbo E, Bipolar Disorder Working Group of the Psychiatric Genomics Consortium (2019). Association of Polygenic Liabilities for Major Depression, Bipolar Disorder, and Schizophrenia With Risk for Depression in the Danish Population. JAMA Psychiatry.

[24] Epic Systems Corporation.

[25] Richter M, Storck M, Blitz R, Goltermann J, Seipp J, Dannlowski U, Baune BT, Dugas M, Opel N Continuous digital collection of patient-reported outcomes during inpatient treatment for affective disorders - implementation and feasibility. MedRxiv..

[26] Geschäftsbericht 2019. UKM.

[27] Qualitätsbericht 2018. UKM.

[28] Geschäftsbereich medizinisches management medizincontrolling. UKM.

[29] Dedalus to acquire Agfa's HCIS business: The signify view. Signify Research.

[30] Operational data model (ODM)-XML. CDISC.

[31] Define-XML. CDISC.

[32] Health Level Seven International (HL7).

[33] NextGen Connect (formerly Mirth Connect).

[34] Java. Oracle.

[35] ECMAScript 6. W3 Schools.

[36] What is TypeScript?. TypeScript.

[37] MongoDB Java drivers. MongoDB.

[38] Json-lib. Sourceforge.

[39] MongoDB.

[40] Docker.

[41] Red Hat.

[42] SPSS statistics. IBM.

[43] Adobe photoshop. Adobe.

[44] Microsoft Visio.

[45] Blitz R, Dugas M (2020). Conceptual Design, Implementation, and Evaluation of Generic and Standard-Compliant Data Transfer into Electronic Health Records. Appl Clin Inform.

[46] Stahl T, Efftinge S, Haase A, Volter M (2012). Modellgetriebene Softwareentwicklung: Techniken, Engineering, Management.

[47] Soto-Rey I, Rehr M, Bruland P, Zeidler C, Riepe C, Steinke S, Ständer S, Dugas M, Storck M (2018). Electronic Collection of Multilingual Patient-Reported Outcomes across Europe. Methods Inf Med.

[48] Dugas Martin, Storck Michael, Soto-Rey (2016). Implementation of an ODM and HL7 Compliant Electronic Patient-Reported Outcome System. Stud Health Technol Inform.

[49] Storck M, Dugas-Breit S, Dugas M, Soto-Rey I (2017). MOPAT@HOME: electronic patient reported outcomes filled out at home, evaluated at the hospital. Studies in Health Technology and Informatics; Volume 244-The Practice of Patient Centered Care-Empowering and Engaging Patients in the Digital Era.

[50] REDCap.

[51] Kannan V, Basit M, Youngblood J (2017). Agile co-development for clinical adoption and adaptation of innovative technologies. Health Innov Point Care Conf.

[52] Beck AT, Steer RA, Brown GK (1996). Manual for the Beck Depression Inventory-II.

[53] Kühner C, Bürger C, Keller F, Hautzinger M (2007). Reliability and validity of the Revised Beck Depression Inventory (BDI-II). Results from German samples. Nervenarzt.

[54] Soto CJ, John OP (2017). Short and extra-short forms of the Big Five Inventory–2: The BFI-2-S and BFI-2-XS. Journal of Research in Personality.

[55] (1994). Childhood trauma questionnaire. APA PsycNet.

[56] Hamilton M (1986). The Hamilton rating scale for depression. Assessment of Depression.

[57] Leckelt M, et al (2017). Supplemental Material for Validation of the Narcissistic Admiration and Rivalry Questionnaire Short Scale (NARQ-S) in Convenience and Representative Samples. Psychological Assessment.

[58] The SCL-90-R and brief symptom inventory, and matching clinical rating scales. APA PsycNet.

[59] JavaScript Object Notation.

[60] JavaScript operational data model. GitHub.

[61] JavaScript Object Notation Schema.

[62] Bargas-Avila J, Brenzikofer O, Roth S, User A (2010). Simple but crucial user interfaces in the world wide web: introducing 20 guidelines for usable web form design. IntechOpen.

[63] Idrus Z, Razak N (2010). Using three layer model (TLM) in web form design: WeFDeC checklist development. ICCEA NTAA.

[64] (2010). Research-based web design and usability guidelines. U.S. Department of Health & Human Services.

[65] Seckler M, Heinz S, Bargas-Avila J (2014). Designing usable web forms: Empirical evaluation of web form improvement guidelines.

[66] Beck depression inventory. Portal für Medizinische Datenmodelle (MDM-Portal).

[67] Big five inventory (BFI-2-S) Big five - SEED studie. Portal für Medizinische Datenmodelle (MDM-Portal).

[68] Big five inventory (BFI-2-XS) - SEED studie. Portal für Medizinische Datenmodelle (MDM-Portal).

[69] Childhood trauma questionnaire (CTQ). Portal für Medizinische Datenmodelle (MDM-Portal).

[70] Family mental history - SEED-studie. Portal für Medizinische Datenmodelle (MDM-Portal).

[71] Worboys M (2013). The Hamilton Rating Scale for Depression: The making of a "gold standard" and the unmaking of a chronic illness, 1960-1980. Chronic Illn.

[72] NARQ-S. Portal für Medizinische Datenmodelle (MDM-Portal).

[73] SCL-90 somatisation scale - SEED-studie. Portal für Medizinische Datenmodelle (MDM-Portal).

[74] Sociodemographic questionnaire - SEED-studie. Portal für Medizinische Datenmodelle (MDM-Portal).

[75] Questions on individual disease course - SEED-studie. Portal für Medizinische Datenmodelle (MDM-Portal).

[76] Questions on somatic comorbidities - SEED-studie. Portal für Medizinische Datenmodelle (MDM-Portal).

[77] Seed clinical data admission date/time. GitHub.

[78] Seed clinical data classification. GitHub.

[79] Seed clinical data DRG/diagnosis. GitHub.

[80] Seed clinical data ECT. GitHub.

[81] Seed Clinical data laboratory assessments. GitHub.

[82] Seed clinical data medication. GitHub.

[83] Seed clinical data patient. GitHub.

[84] Seed clinical data vital signs. GitHub.

[85] Kim E, Rubinstein SM, Nead KT, Wojcieszynski AP, Gabriel PE, Warner JL (2019). The Evolving Use of Electronic Health Records (EHR) for Research. Semin Radiat Oncol.

[86] Skyttberg N, Chen R, Koch S (2018). Man vs machine in emergency medicine - a study on the effects of manual and automatic vital sign documentation on data quality and perceived workload, using observational paired sample data and questionnaires. BMC Emerg Med.

[87] Butler A, Wei W, Yuan C, Kang T, Si Y, Weng C (2018). The Data Gap in the EHR for Clinical Research Eligibility Screening. AMIA Jt Summits Transl Sci Proc.

[88] FDA resources for data standards. US Food and Drug Administration.

[89] Genomics England uses MongoDB to power the data science behind the 100,000 genomes project. MongoDB.

[90] Esteva A, Kuprel B, Novoa RA, Ko J, Swetter SM, Blau HM, Thrun S (2017). Dermatologist-level classification of skin cancer with deep neural networks. Nature.

[91] Kambeitz J, Cabral C, Sacchet MD, Gotlib IH, Zahn R, Serpa MH, Walter M, Falkai P, Koutsouleris N (2017). Detecting Neuroimaging Biomarkers for Depression: A Meta-analysis of Multivariate Pattern Recognition Studies. Biol Psychiatry.

[92] Cearns M, Opel N, Clark S, Kaehler C, Thalamuthu A, Heindel W, Winter T, Teismann H, Minnerup H, Dannlowski U, Berger K, Baune BT (2019). Predicting rehospitalization within 2 years of initial patient admission for a major depressive episode: a multimodal machine learning approach. Transl Psychiatry.

[93] Koutsouleris N, Dwyer DB, Degenhardt F, Maj C, Urquijo-Castro MF, Sanfelici R, Popovic D, Oeztuerk O, Haas SS, Weiske J, Ruef A, Kambeitz-Ilankovic L, Antonucci LA, Neufang S, Schmidt-Kraepelin C, Ruhrmann S, Penzel N, Kambeitz J, Haidl TK, Rosen M, Chisholm K, Riecher-Rössler Anita, Egloff L, Schmidt A, Andreou C, Hietala J, Schirmer T, Romer G, Walger P, Franscini M, Traber-Walker N, Schimmelmann BG, Flückiger Rahel, Michel C, Rössler Wulf, Borisov O, Krawitz PM, Heekeren K, Buechler R, Pantelis C, Falkai P, Salokangas RKR, Lencer R, Bertolino A, Borgwardt S, Noethen M, Brambilla P, Wood SJ, Upthegrove R, Schultze-Lutter F, Theodoridou A, Meisenzahl E, PRONIA Consortium (2021). Multimodal Machine Learning Workflows for Prediction of Psychosis in Patients With Clinical High-Risk Syndromes and Recent-Onset Depression. JAMA Psychiatry.

[94] Khalilia M, Choi M, Henderson A, Iyengar S, Braunstein M, Sun J (2015). Clinical Predictive Modeling Development and Deployment through FHIR Web Services. AMIA Annu Symp Proc.

[95] OMOP data model. Observational Medical Outcomes Partnership.

[96] Fusar-Poli P, Oliver D, Spada G, Patel R, Stewart R, Dobson R, McGuire P (2019). Real World Implementation of a Transdiagnostic Risk Calculator for the Automatic Detection of Individuals at Risk of Psychosis in Clinical Routine: Study Protocol. Front Psychiatry.

[97] NIH common data elements repository. National Library of Medicine.

[98] Systematized Nomenclature of Medicine.

[99] Unified Medical Language System.

[100] Seed project. GitHub.

[101] Hallensleben N, Glaesmer H, Forkmann T, Rath D, Strauss M, Kersting A, Spangenberg L (2019). Predicting suicidal ideation by interpersonal variables, hopelessness and depression in real-time. An ecological momentary assessment study in psychiatric inpatients with depression. Eur Psychiatry.

