# Supplementary figures and images for: Design and Implementation of an Informatics Infrastructure for Standardized Data Acquisition, Transfer, Storage, and Export in Psychiatric Clinical Routine: Feasibility Study
Source: JMIR Ment Health. 2021 Jun 9;8(6):e26681. doi: 10.2196/26681 (PMC8262601; doi:10.2196/26681)

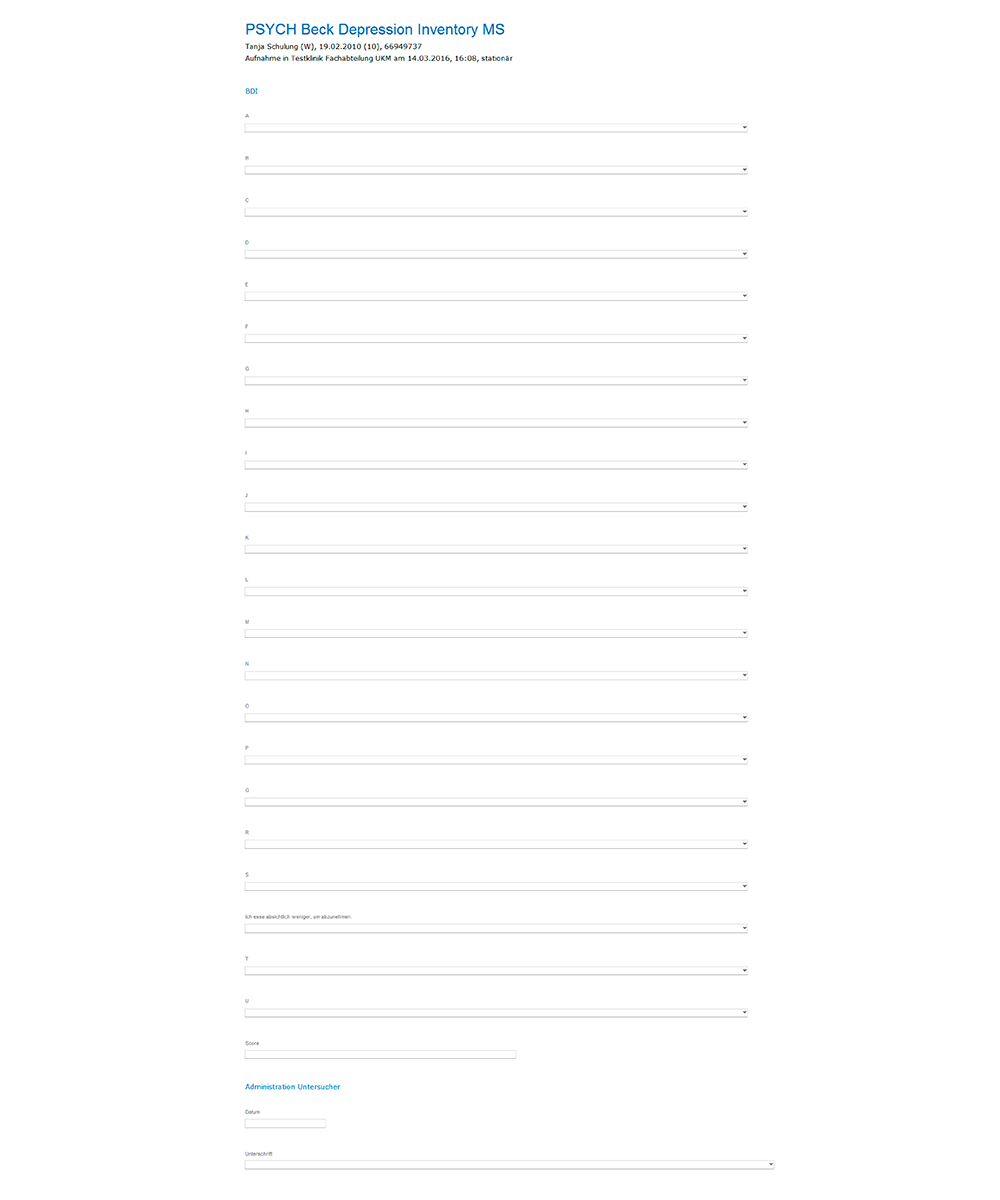

Supplement: Multimedia Appendix 1 [file mental_v8i6e26681_app1.png]

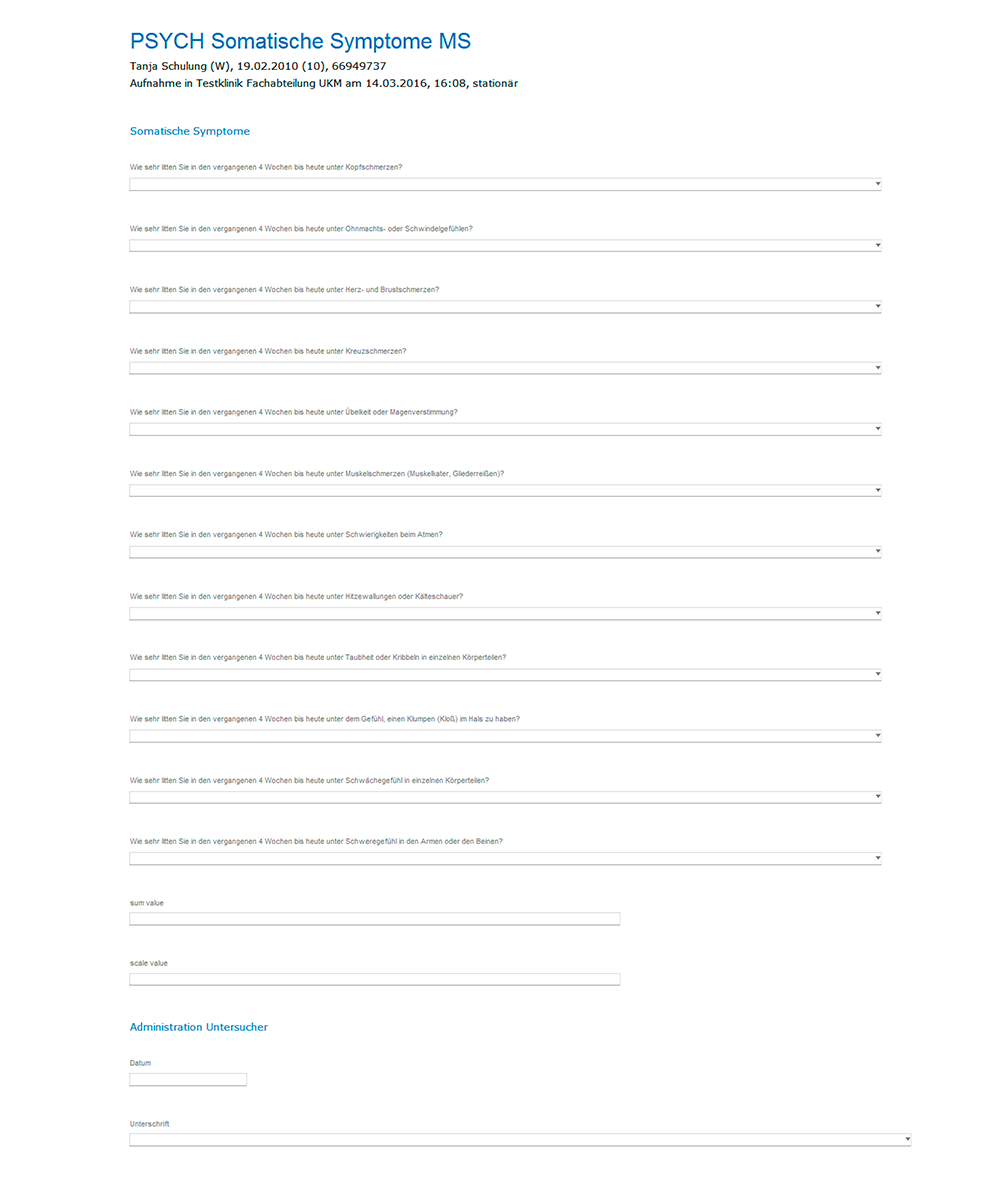

Supplement: Multimedia Appendix 2 [file mental_v8i6e26681_app2.png]
